# Road Traffic Accident among Patients Presenting to the Emergency Department of a Tertiary Care Centre: A Descriptive Cross-sectional Study

**DOI:** 10.31729/jnma.8032

**Published:** 2023-02-28

**Authors:** Manohar Pradhan, Hari Prasad Upadhyay, Ayasha Shrestha, Alok Pradhan

**Affiliations:** 1 Department of General Practice and Emergency Medicine, College of Medical Sciences, Bharatpur, Chitwan, Nepal; 2 Department of Community Medicine, College of Medical Sciences, Bharatpur, Chitwan, Nepal

**Keywords:** *emergencies*, *mortality*, *soft tissue injury*, *traffic accidents*

## Abstract

**Introduction:**

Road traffic accidents are a public health problem and have emerged as the leading cause of mortality and morbidity. Head is the most commonly affected site of road traffic accidents. The aim of this study was to find out the prevalence of road traffic accidents among patients presenting to the emergency department of a tertiary care centre.

**Methods:**

A descriptive cross-sectional study was conducted at the Emergency Department from 12 January 2022 to 14 June 2022. Ethical approval was taken from the Institutional Review Committee (Reference number: COMSTH-IRC/2021-171). Data was collected using a self-structure questionnaire and from emergency tickets. A convenience sampling method was used. Point prevalence and 95% Confidence Interval were calculated.

**Results:**

Among 7654 patients, the prevalence of road traffic accidents was found to be 734 (9.58%) (8.49-10.66, 95% Confidence Interval). Most of the accidents took place on Friday 139 (18.94%). The majority of them were soft tissue injuries 279 (38.01%).

**Conclusions:**

The prevalence of road traffic accidents was found to be higher compared to similar studies done in similar settings. Accident preventive strategies should be focused on and implemented by all the stakeholders.

## INTRODUCTION

Road traffic accidents (RTA) account for a significant proportion of unintentional injuries.^1^ In the past two decades, countries have seen significant growth in urbanisation, motorization, industrialization, and changes in the socioeconomic level of the societies.^2^ Also, RTA has increased and it has now become a major cause of disability and death globally.^3^

Road injuries due to road traffic accidents are a major public health problem in most developing and developed countries. RTAs are increasing day by day leading to injuries, disabilities and deaths.^4^ The most common risk factors associated with RTA are over speed, driving under influence, not using safety measures such as seat belts, helmets, and child restraints, poorly constructed roads, increased number of vehicles that are poorly maintained, unplanned urbanization and industrialization, motorization, overpopulation and fragile traffic rules.^5,6^

The aim of this study was to find out the prevalence of road traffic accidents among patients presenting to the emergency department of a tertiary care centre.

## METHODS

This descriptive cross-sectional study was conducted at the Emergency Department of the College of Medical Sciences and Teaching Hospital from 12 January 2022 to 14 June 2022. Ethical approval was taken from the Institutional Review Committee (Reference number: COMSTH-IRC/2021-171). All the patients who had given consent for data collection with road traffic accidents were included in this study. Patients were selected by using a convenience sampling method. The sample size was calculated using the following formula:


$$\begin{array}{ll} \text{n} & ={\text{Z}}^{2}\times \frac{\text{p}\times \text{q}}{{\text{e}}^{\text{2}}}\\ & =1.96^{2}\times \frac{0.50\times 0.50}{0.04^{2}}\\ & =601 \end{array}$$

Where,

n = minimum required sample size
Z = 1.96 at a 95% Confidence Interval (CI)
p = prevalence of taken as 50% for maximum sample size calculation
q = 1-p
e = margin of error, 4%

The minimum sample size calculated was 601. However, the final sample size taken was 734.

Data were collected using a self-structure questionnaire and from emergency tickets. Sociodemographic data were recorded from emergency tickets while other information was asked of patients/visitors. A systematic medical examination was done and recorded in the questionnaire using a pre-designed questionnaire and then collected data were checked for completeness and accuracy.

Data collected were entered and analysed using IBM SPSS Statistics version 20. Point estimate and 95% CI were calculated.

## RESULTS

Among 7654 patients, road traffic accidents was found to be 734 (9.58%) (8.49-10.66, 95% CI). The majority 308 (41.96%) patients were from the 20-30 years age group followed by 132 (17.98%) patients from 3040 years of age group. The mean age were found to be 25.18±4.85 years. Among them, the majority were males 456 (62.13%). The majority had studied up to higher secondary level education status, 184 (25%). Most of them were students 242 (32.97%) and belonged to joint families 624 (85.01%) and most of them were married 448 (61.04%) (Table 1).

**Table 1:** Sociodemographic characteristics of RTA patients (n= 734).

Variables	n (%)
**Age (years)**	
<10	59 (8.04)
10-20	125 (17.03)
20-30	308 (41.96)
30-40	132 (17.98)
40-50	44 (5.99)
50-60	29 (3.95)
>60	37 (5.04)
**Sex**	
Male	456 (62.13)
Female	278 (37.87)
**Education status**	
Illiterate	110 (14.99)
Primary	73 (9.95)
Lower secondary	88 (11.99)
Secondary	125 (17.03)
Higher secondary	184 (25.07)
Bachelor and above	154 (20.98)
**Occupation**	
Student	242 (32.97)
Business	110 (14.99)
Agriculture	176 (23.98)
Labor	103 (14.04)
Service	81 (11.04)
Not known	23 (3.13)
**Marital status**	
Single	286 (38.96)
Married	448 (61.04)
**Type of family**	
Nuclear	110 (14.99)
Joint	624 (85.01)

Most of the accidents took place on Friday 139 (18.94%) with least on Tuesday 74 (10.08%). A total of 301 (41.01%) cases of RTA took place in the daytime from 12-6 PM followed by 228 (31.06%) in 6 PM-12 AM (Table 2).

**Table 2:** Details involved in the accident (n= 734).

Variables	n (%)
**Day of the accident**	
Sunday	93 (12.67)
Monday	102 (13.90)
Tuesday	74 (10.08)
Wednesday	84 (11.44)
Thursday	116 (15.80)
Friday	139 (18.94)
Saturday	125 (17.03)
**Time of accident**	
12-6 AM	73 (9.95)
6 AM-12 PM	132 (17.98)
12-6 PM	301 (41.01)
6 PM-12 AM	228 (31.06)
**Site of accident**	
Highway	477 (64.99)
Lanes	73 (10)
Road	184 (25.07)
**Type of vehicle involved**	
2 wheelers	147 (20.03)
3 wheelers	110 (14.99)
4 wheelers	477 (64.99)

Collision between vehicles 352 (47.96%) was found to be the major type of accident. Soft tissue injuries was seen in 279 (38.01%) followed by head injuries in 228 (31.06%) cases. A total of 301 (41.01%) patients were referred to general ward and 284 (38.69%) were discharged (Table 3).

**Table 3:** Nature, type of injuries and referral of road traffic accident (n= 734).

Variables	n (%)
**Nature of accident**	
Hit by the vehicle	242 (32.97)
Collison of vehicle	352 (47.961
Overturning of vehicle	95 (12.94)
Fall from moving vehicle	44 (5.99)
**Type of Injury**	
Head injury	228 (31.06)
Spine injury	11 (1.50)
Chest trauma	51 (6.95)
Fracture of limb bone	120(16.35)
Pelvic injury	22 (3.00)
Soft tissue injures (except scalp injury)	279 (38.01)
Brought dead	9(1.23)
Others	15(2.04)
**Patients referred**	
ICU	88(11.99)
General ward	301 (41.01)
Operation theatre	33 (4.50)
Died in ER	19(2.59)
Brought dead	9(1.23)
Discharge	284 (38.69)

## DISCUSSION

The prevalence of road traffic accident was found to be 9.58% which was higher compared to other studies conducted in Eastern part of Nepal where the prevalence of RTA was reported to be 4% whereas the study conducted in India reported the prevalence to be 41.4%.^7,8^ In a similar study, the prevalence of RTA was reported to be 33.2% with 77% male.^9^ In our study also, the majority of the RTA victims were male (62.13%) and between 20-30 years (41.96%). A similar study conducted in India showed that 75% of the patients were less than 40 years.^10^ In a similar study it was reported that (98.90%) were males and (52.50%) of cases were belonging to the age group of 21-30 years.^11^ This may be due to the involvement of male in their outdoor work or their involvement in violent activities as compared to female. Furthermore, the majority of victims were educated up to a Higher secondary level (25.07%) followed by bachelor and above (20.98%). Similarly, one study from India showed that the majority of the cases (39.3%) were graduates, followed by (31.1%) with intermediate education and (11.5%) were illiterate.^11^

Majority of the victims were students (32.97%) followed by agricultural workers (23.98%). Similar findings were seen in a study conducted in the Eastern part of Nepal.^7^ Similarly, the majority of them were married (61.04%) and belonged to a joint family (85.01%). Similar findings were seen in a study done in the western part of Nepal.^12^ The distribution of weekdays shows a denser cluster of accidents on Friday (18.94%) and Saturday (17.03%), which are weekends in Nepal. It could be the higher proportion of the people's movement for marketing and outing on weekends. Another study also reported that weekends were the most common time for accidents.^13^ In our study it was found that the majority of RTA took place in the daytime from 12-6 PM. Similar trends were also observed in other studies conducted in various parts of Nepal and India.^14^ These hours are the busiest as commuters go to and return from the schools, offices, factories and business places. It could also be due to stress and fatigued drivers because of continuous work and visibility which was reported in studies done in China and Croatia.^15^

Majority (64.99%) of accidents were in the highway areas. In a similar study done in Nepal, similar findings were seen.^16^ Nearly half of the respondents thus stated that the mechanism of the incident was due to the collision of vehicles (47.96%) and it was mostly four-wheelers (5.99%). The findings reported were different from another study in Nepal and various parts of India.^17^

Majority of the RTA was due to personal causes (43.05%). A similar finding was also seen in study.^17^ Considering all the cases of injuries, the majority was soft-tissue injury (38.01%) followed by head injuries (31%) and nearly half of the patients were referred to the General ward (41.01%) followed by discharge (38.69%). In a similar study conducted in Nepal, the most common time duration of occurrence of RTA was reported to be 6-12 PM (62%), most common injury after RTA was head injury 64%.^7^ These similar findings were seen in a study conducted at Karnali.^19^ Among all the injured patients, 3.80% were dead. Mortality among RTA cases was found to be 3.15%.^7^ In another study, similar findings were seen.^18^

The present study was a descriptive cross-sectional study conducted in a single centre, the result may not be generalised to other settings of Nepal. Data was collected from the patient's side which might have information and response bias.

## CONCLUSIONS

The prevalence of road traffic accidents was found to be higher compared to similar studies done in similar settings. Young adult males with higher secondary education become more victims of road traffic accidents. Soft tissue injuries and head injuries were the commonest sites of injury in these areas. This shows that understanding such factors are crucial in developing and implementing accident prevention strategies in present scenarios.
